# Detection of SARS-COV-2 variants and their proportions in wastewater samples using next-generation sequencing in Finland

**DOI:** 10.1038/s41598-024-58113-8

**Published:** 2024-04-02

**Authors:** Anssi Lipponen, Aleksi Kolehmainen, Sami Oikarinen, Anna-Maria Hokajärvi, Kirsi-Maarit Lehto, Annamari Heikinheimo, Jani Halkilahti, Aapo Juutinen, Oskari Luomala, Teemu Smura, Kirsi Liitsola, Soile Blomqvist, Carita Savolainen-Kopra, Tarja Pitkänen, WastPan Study Group, Annika Länsivaara, Rafiqul Hyder, Erja Janhonen, Annamari Heikinheimo, Viivi Heljanko, Venla Johansson, Paula Kurittu, Ananda Tiwari, Ahmad Al-Mustapha, Anniina Sarekoski, Teemu Möttönen, Kati Räisänen, Carita Savolainen-Kopra

**Affiliations:** 1 Expert Microbiology Unit, Department of Health Security, Finnish Institute for Health and Welfare, Kuopio, Finland; 2 Institute of Biomedicine, Faculty of Health Sciences, University of Eastern Finland, Kuopio, Finland; 3 Faculty of Medicine and Health Technology, Tampere University, Tampere, Finland; 4 Department of Food Hygiene and Environmental Health, Faculty of Veterinary Medicine, University of Helsinki, Helsinki, Finland; 5 Microbiology Unit, Laboratory and Research Division, Finnish Food Authority, Helsinki, Finland; 6 Expert Microbiology Unit, Department of Health Security, Finnish Institute for Health and Welfare, Helsinki, Finland; 7 Infectious Disease Control and Vaccinations Unit, Department of Health Security, Finnish Institute for Health and Welfare, Helsinki, Finland; 8 Department of Virology, Faculty of Medicine, University of Helsinki, Helsinki, Finland

**Keywords:** SARS-CoV-2, Water microbiology, Environmental microbiology

## Abstract

Severe Acute Respiratory Syndrome Coronavirus 2 (SARS-CoV-2) variants may have different characteristics, e.g., in transmission, mortality, and the effectiveness of vaccines, indicating the importance of variant detection at the population level. Wastewater-based surveillance of SARS-CoV-2 RNA fragments has been shown to be an effective way to monitor the COVID-19 pandemic at the population level. Wastewater is a complex sample matrix affected by environmental factors and PCR inhibitors, causing insufficient coverage in sequencing, for example. Subsequently, results where part of the genome does not have sufficient coverage are not uncommon. To identify variants and their proportions in wastewater over time, we utilized next-generation sequencing with the ARTIC Network's primer set and bioinformatics pipeline to evaluate the presence of variants in partial genome data. Based on the wastewater data from November 2021 to February 2022, the Delta variant was dominant until mid-December in Helsinki, Finland’s capital, and thereafter in late December 2022 Omicron became the most common variant. At the same time, the Omicron variant of SARS-CoV-2 outcompeted the previous Delta variant in Finland in new COVID-19 cases. The SARS-CoV-2 variant findings from wastewater are in agreement with the variant information obtained from the patient samples when visually comparing trends in the sewerage network area. This indicates that the sequencing of wastewater is an effective way to monitor temporal and spatial trends of SARS-CoV-2 variants at the population level.

## Introduction

Severe Acute Respiratory Syndrome Coronavirus 2 (SARS-CoV-2) has caused a worldwide pandemic with severe socio-economic impacts, indicating the importance of monitoring this pandemic at the population level^1^. SARS-CoV-2 variants may have different characteristics, e.g., in transmission, mortality, and the effectiveness of vaccines, indicating the importance of variant detection at the population level^2^.

Recently, it has been reported that subvariants of SARS-CoV-2 are escaping neutralizing antibodies, causing risk of reinfection and indicating the importance of variant tracing^3^. Wastewater-based surveillance of SARS-CoV-2 RNA fragments has been shown to be an effective way to monitor COVID-19 pandemic trends at the population level^4^. Wastewater-based surveillance has also indicated the potential to detect mutations that have not been detected in clinical samples, and so it could be a valuable complement for clinical surveillance^5,6^.

SARS-CoV-2 variants have been detected from wastewater by using next-generation sequencing widely over the world, e.g., in Europe^7–9^ as well as in North and South America^10,11^. Also, various NGS library methods such as enrichment panels^12^, amplicon panels^11,13^, and metagenomic shotgun sequencing^12^ have been utilized. Frequently, part of the genome does not have sufficient coverage, which may be related to wastewater being a complex sample matrix containing environmental factors and PCR inhibitors, possibly leading to the coverage problem^14^. This and a highly variable number of variant-defining mutations between known SARS-CoV-2 variants makes variant detection in wastewater samples difficult, especially when part of the variant-defining mutations are in the genomic region with low or null coverage.

In this study, we present next-generation sequencing utilizing the ARTIC Network’s primer set, including the bioinformatics pipeline to follow temporal and spatial trends of SARS-CoV-2 variants in Finland. The pipeline outcome includes (1) sequence quality control with parameters of uniformity, coverage, and the number of mapped reads, (2) a probability calculation with a hypergeometric test to evaluate the existence of a variant, even if part of the genome is not covered, and (3) the evaluation of proportions of SARS-CoV-2 variants in wastewater.

## Materials and methods

### Wastewater samples

**Figure 1:**
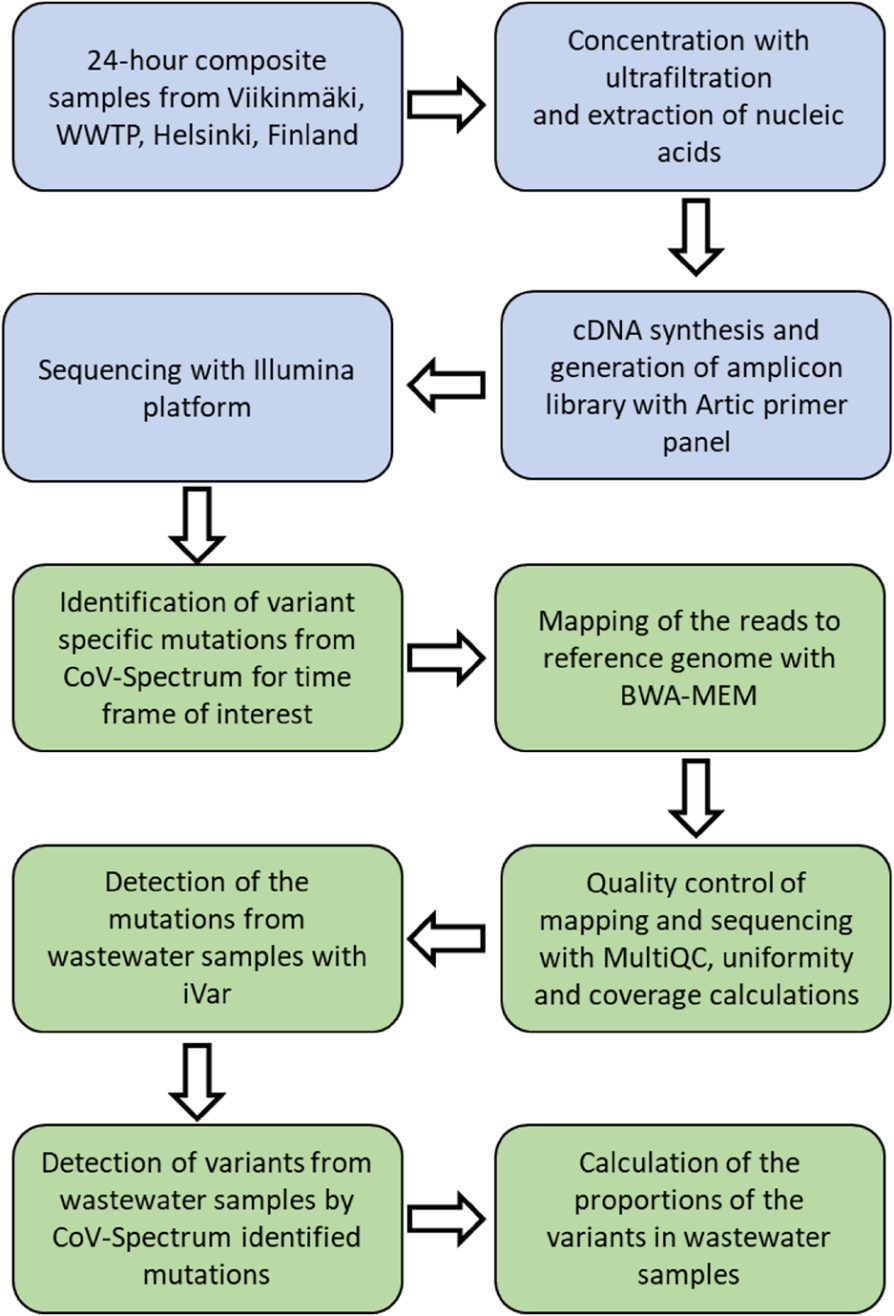
Flowchart presenting the pipeline to identify SARS-CoV-2 variants from wastewater in Finland. The blue boxes present the laboratory tasks and the green boxes bioinformatics tasks.

For the purpose of variant detection, 24-h composite wastewater samples were collected, as described in Hokajärvi et al.^15^ and delivered within 24 h to a laboratory for analysis. Sixteen influent wastewater samples from 8 November 2021 to 28 February 2022 were collected weekly, following the standard biosafety precautions for handling untreated wastewater, from Viikinmäki Wastewater Treatment Plant (WWTP) in Helsinki, Finland. Viikinmäki WWTP serves the municipalities of Helsinki, Sipoo, Kerava, Tuusula, Järvenpää, Pornainen, Mäntsälä, and partly also Vantaa, covering a total population of 860 000 inhabitants. The steps of the analysis pipeline starting from wastewater samples collected from the wastewater treatment plant are summarized in Fig. 1.

### Extraction of nucleic acids

Nucleic acids were extracted from wastewater, as described in Hokajärvi et al. and Tiwari et al*.*^15,16^. In brief, 70 ml of wastewater was pre-centrifugated and supernatants were concentrated with Centricon Plus-70 centrifugal filters (#UFC701008, Merck, Germany) with a concentration time of 25 min in 3000*g*, producing 240–930 µl of concentrate. The concentrate volume less than 700 µl was normalized to 700 μl for all filtrated supernatants with flow-through wastewater. Nucleic acid was extracted from 300 µl of concentrate using a Chemagic-360D (Perkin-Elmer, MA, USA) instrument with a Chemagic Viral300 DNA/RNA extraction kit (#CMG-1033-S, Perkin-Elmer), and eluted to a 50- or 60-µl kit elution buffer.

### Generation of amplicon libraries and next-generation sequencing

Nucleic acids extracted from wastewater samples may contain PCR inhibitors causing poor assay sensitivity, which also need to be taken into account in NGS-based methods^14^. To maximize the odds of ensuring high-quality sequencing data by minimizing the risk of inhibition, undiluted and diluted (1:5 or 1:10) RNA was used for cDNA synthesis with LunaScript RT SuperMix (#E3010L, New England Biolabs, MA, USA) in 20-µl reaction volume in accordance with the manufacturer’s protocol.

To generate the amplicon sequencing library, multiplex PCR was performed by using ARTIC.v4 (samples from 8 and 21 November 2021) or ARTIC.v4.1 primers (samples for any other date)^17^. An ARTIC Illumina library constructed by using 15 µl of amplified cDNA was purified with QuickStep™ 2 SOPE Resin and EdgeBio Optima DTR 96-well Plate by Edge Biosystems (Edge Biosystems Inc, CA, USA). Library preparation was performed in accordance with COVID-19 ARTIC v3 Illumina library construction and sequencing protocol v.4. Library preparation was miniaturized to × 0.25 from the original reaction volume. Unique Dual Index UMI oligos by IDT (Integrated DNA Technologies, IA, USA) were used as ligation adapters. Illumina-specific p5 and p7 primers were introduced in library amplification PCR. An equivolume pool was formed from amplified libraries and purified from adapter-dimers using Agencourt AMPure XP SPRI paramagnetic bead chemistry (Beckman Coulter, Indianapolis, IN, USA). The library pool was quantified for sequencing using LabChip GX Touch HT High Sensitivity assay (PerkinElmer, Waltham, MA, USA). Sequencing was performed with the Illumina NovaSeq 6000 system using an SP flow cell with a lane divider (Illumina, San Diego, CA, USA) with paired-end 251 bp reads.

### Identification of variant specific mutations

Specific mutations of the variants were identified from a CoV-Spectrum platform (https://cov-spectrum.org) based on the Global Initiative on Sharing All Influenza Data (GISAID)^18,19^. From CoV-Spectrum, lists of mutations and mutation frequencies in variant sequences of each variant likely to be present in the population—in this case Delta, Omicron BA.1, and Omicron BA.2—were downloaded. Using in-house R-script, mutations that were present in more than 80% of the sequences were identified and then compared to mutations of other variants. Only mutations unique to each variant were left on the mutation reference list.

To use this pipeline in other time frames of interest, a new list of variant specific mutations would need to be generated. In addition, other strategies for generating the reference list can be used, for example including mutations which might affect transmission or disease severity.

### Mapping of the reads and quality control

Reads were mapped to reference the SARS-CoV-2 genome (NC_045512.2) with the Burrows-Wheeler Alignment Tool (BWA-MEM)^20^ using the default settings. Quality control of the raw reads and mapped reads was performed using FastQC and MultiQC^21,22^. To evaluate the quality of sequencing and mapping to the reference genome, we calculated the on-target coverage percentage of each nucleotide in the sample when the depth of reads was > 100 and > 1, as well as uniformity. Uniformity was calculated thus:

$$Uniformity=\frac{Total\;number\;of\;bases\;with\;coverage\ge 20\%\;of\;mean}{Total\;number\;of\;targeted\;bases}$$

For further analysis, sequencing data from RNA dilution producing the highest uniformity was used.

### Identification of mutations from wastewater samples

ARTIC primers were trimmed from the BAM file with iVar using the *ivar trim* command, using the default settings (min-length 30; min-quality 20; sliding-window-width 4)^23^. Mutations, deletions, and insertions were identified from the trimmed BAM file with *ivar variants* by using the default settings (minimum quality 20; minimum frequency threshold 0.03, minimum depth 0)^23,24^.

By means of iVar output filing, mutations in the samples were divided into three categories:

- SARS-CoV-2 detected in the sample: > 20 reads were mapped across the reference genome (NC_045512.2).
- Mutation in SARS-CoV-2 in a specific locus can be detected: > 20 read mapped mutation reads per nucleotide^25^.
- Allelic frequency of the SARS-CoV-2 variants in the sample can be calculated: > 20 mutation reads, coverage > 100 mapped reads per nucleotide, and the p-value of a Fisher’s exact test from iVar for mutations < 0.05, which ensure that allelic frequency in a given position is higher than the mean error rate^23^.

Since the capability to detect mutations in the sample may vary in accordance with sequencing coverage and uniformity of the reads across the genome, and so all the nucleotides of the genome may not be sequenced as high coverage, a hypergeometric test was applied to justify the existence of the variant in the sample. The hypergeometric test was performed using the *phyper* function in R (version 4.3.1), utilizing the total number of mutations in the sample, the total number of variant specific mutations found in the sample, the total number of variant specific mutations in the reference, and the total number of nonspecific mutations found in the sample. Based on the results, the variants with the p-value < 0.05 were considered detected.

### Proportions of variants in wastewater

To define proportions of the SARS-CoV-2 variants in the sample, average and standard deviation from the nucleotide allelic frequencies were calculated from the samples and variant specific nucleotide mutations, which fulfilled the allelic frequency calculation criteria described above.

## Results

### SARS-CoV-2 genomic coverage of sequenced samples

**Figure 2:**
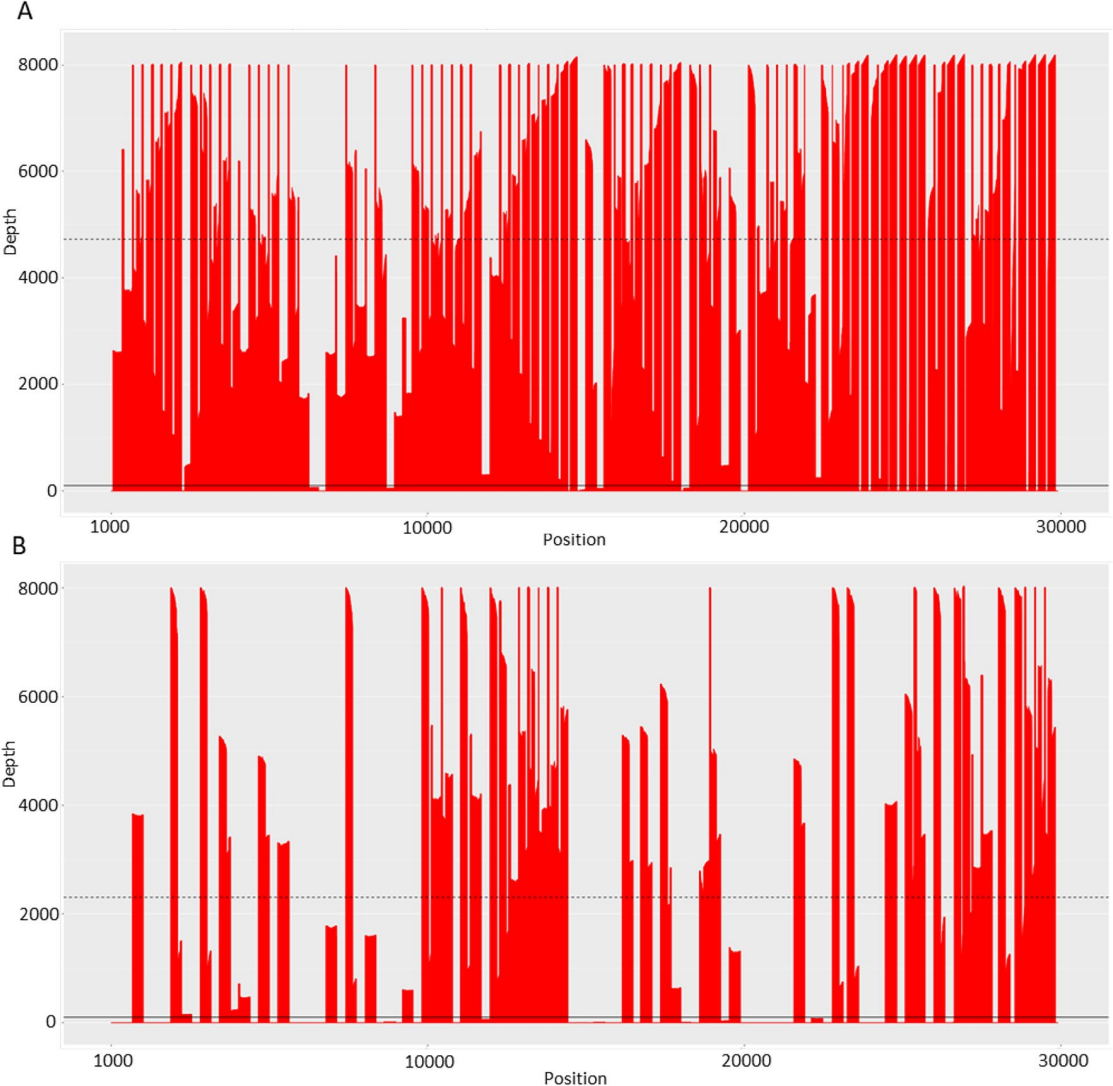
Coverage plots of one sample with two RNA dilution before cDNA synthesis. On Y-axis depth and X-axis SARS-CoV-2 genomic location. (**A**) Sample from 31 January 2022 in which undiluted RNA was used for cDNA synthesis. Coverage 90.0%, uniformity 85.5%, and 5.40 M mapped reads. (**B**) Sample from 31 January 2022 in which 1:5 diluted RNA was used for cDNA synthesis. Coverage 54.3%, uniformity 51.9%, and 1.18 M mapped reads (solid line) indicated a depth of 100 and dashed-line median of the coverage. As coverage in some parts of the genome was less than 100× (area below the black line), the proportions of mutations could not be reliably detected.

Variation in the genomic coverage, uniformity, and number of mapped reads between sequenced samples was observed (Fig. 2). The median of genomic coverage when the depth was > 100 reads per nucleotide was 92.0% (range 66.3–97.1%), the mean of uniformity was 85.5% (range 72.2–90.9%), and the mean of mapped reads 1.916 M (range 0.032–5.403 M) when the highest coverage from two sample dilutions of the same sample was used to identify variants from the given sample. As genomic coverage in some of the samples was < 100%, not all potential mutations could be reliably identified (Table 1**, **Fig. 2).

**Table 1:** Quality parameters of Artic amplicon sequencing and mapping of the reads to SARS-CoV-2 reference genome. The best result by coverage from two sequenced nucleic acid dilutions is presented in the table. Data from Viikinmäki Wastewater Treatment Plant, Helsinki, Finland.

Sampling date	Sample dilution	Uniformity %	Coverage % > 100 reads per nucleotide	Coverage % > 1 reads per nucleotide	Mapped reads (M)
8.11.2021	Undiluted	76.9	89.0	97.0	0.56
15.11.2021	1:5	76.7	89.0	97.5	0.59
22.11.2021	Undiluted	86.8	94.8	97.1	1.23
29.11.2021	1:5	77.9	92.0	98.8	1.91
7.12.2021	1:5	75.2	86.5	97.2	1.13
12.12.2021	1:5	84.2	94.8	99.6	3.79
20.12.2021	1:5	87.8	95.5	99.4	4.16
3.1.2022	1:5	90.9	96.6	99.6	2.74
10.1.2022	Undiluted	88.8	95.8	99.6	0.66
17.1.2022	1:5	89.3	97.1	98.8	2.67
24.1.2022	1:5	88.3	96.1	98.8	3.23
31.1.2022	Undiluted	85.5	90.0	97.9	5.40
7.2.2022	1:5	75.7	80.1	88.1	1.96
14.2.2022	1:10	85.6	96.0	98.8	0.92
21.2.2022	1:5	86.3	67.0	96.4	0.033

### Identification of variant specific mutations from CoV-Spectrum

SARS-CoV-2 variant-specific mutations were identified from the CoV-Spectrum platform (cov-spectrum.org [accessed 24.03.2023]), which is based on sequences of SARS-CoV-2 clinical samples in GISAID^18^. Variant specific mutations were used to recognize SARS-CoV-2 variants from wastewater. To use this pipeline in other time frames where some other variants are present, the list of reference needs to be updated accordingly.

For Delta B.1.617.2, 33 variant specific nucleotide mutations were identified. In total, 21 of these were nucleotide substitutions, of which 18 led to change of amino acid, two were synonymous, and one was an intergenic mutation. A total of 12 nucleotide deletions were identified, from which 11 led to the deletion of amino acids D119-, F120-, E156-, and F157-, and one nucleotide deletion was identified as an amino acid mutation (Table 2).

**Table 2:** Variant specific mutations identified for Delta B.1.617.2, Omicron BA.1, and Omicron BA.2 variants of the SARS-CoV-2 CoV-Spectrum platform, used to identify variants from wastewater samples.

Delta B.1.617.2			Omicron BA.1			Omicron BA.2		
Nucleotide location	Amino acid mutation	Gene	Nucleotide location	Amino acid mutation	Gene	Nucleotide location	Amino acid mutation	Gene
G210T	–	–	A11537G	I3758V	ORF1a	G29744-	–	–
C8986T	–	ORF1a	A26530G	D3G	M	A29740-	–	–
A11332G	–	ORF1a	A2832G	K856R	ORF1a	C29741-	–	–
C25469T	S26L	ORF3a	G6513-	S2083-	ORF1a	C29743-	–	–
T27638C	V82A	ORF7a	T6514-	S2083-	ORF1a	T29748-	–	–
C27752T	T120I	ORF7a	T6515-	L2084I	ORF1a	A29749-	–	–
C27874T	T40I	ORF7b	G8393A	A2710T	ORF1a	C29750-	–	–
G28248-	D119-	ORF8	T11285-	L3674-	ORF1a	G29751-	–	–
A28249-	D119-	ORF8	T11286-	L3674-	ORF1a	A29752-	–	–
T28250-	D119-	ORF8	G11287-	L3674-	ORF1a	T29753-	–	–
T28251-	F120-	ORF8	T5386G	–	ORF1a	C29754-	–	–
T28252-	F120-	ORF8	T13195C	–	ORF1a	G29755-	–	–
C28253-	F120-	ORF8	C15240T	–	ORF1a	A29756-	–	–
A22029-	E156-	S	A27259C	–	ORF6	G29757-	–	–
G22030-	E156-	S	T22196-	L212I	S	G29745-	–	–
T22031-	F157-	S	T22673C	S371L	S	A29746-	–	–
T22032-	F157-	S	G22898A	G446S	S	G29747-	–	–
C22033-	F157-	S	G23048A	G496S	S	A9424G	–	ORF1a
A22034-	R158G	S	C23202A	T547K	S	C10198T	–	ORF1a
T22917G	L452R	S	C24130A	N856K	S	G10447A	–	ORF1a
G24410A	D950N	S	C24503T	L981F	S	C12880T	–	ORF1a
G29402T	D377Y	N	C21762T	A67V	S	C15714T	–	ORF1a
G4181T	A1306S	ORF1a	C21767-	H69-	S	A20055G	–	ORF1a
C6402T	P2046L	ORF1a	A21768-	H69-	S	C26858T	–	M
C7124T	P2287S	ORF1a	T21769-	H69-	S	A29510C	S413R	N
G9053T	V2930L	ORF1a	G21770-	V70-	S	T670G	S135R	ORF1a
A11201G	T3646A	ORF1a	C21846T	T95I	S	C2790T	T842I	ORF1a
G15451A	G662S	ORF1b	T21988-	G142-	S	G4184A	G1307S	ORF1a
C16466T	P1000L	ORF1b	G21989-	V143-	S	C9344T	L3027F	ORF1a
C19220T	A1918V	ORF1b	T21990-	V143-	S	C9534T	T3090I	ORF1a
T26767C	I82T	M	T21991-	V143-	S	C9866T	L3201F	ORF1a
A28461G	D63G	N	T21992-	Y144-	S	T11294-	F3677-	ORF1a
G28916T	G215C	N	A21993-	Y144-	S	T11295-	F3677-	ORF1a
			T21994-	Y144-	S	T11296-	F3677-	ORF1a
			T21995-	Y145D	S	C17410T	R1315C	ORF1b
			A22194-	N211-	S	C19955T	T2163I	ORF1b
			T22195-	N211-	S	C26060T	T223I	ORF3a
						G27382C	D61L	ORF6
						A27383T	D61L	ORF6
						T27384C	D61L	ORF6
						T21633-	L24-	S
						A21634-	L24-	S
						C21635-	P25-	S
						C21636-	P25-	S
						C21637-	P25-	S
						C21638-	P26-	S
						C21639-	P26-	S
						T21640-	P26-	S
						G21641-	A27S	S
						T22200G	V213G	S
						A22688G	T376A	S
						G22775A	D405N	S
						A22786C	R408S	S

In the Amino Acid mutation column, - indicates that nucleotide mutation is in the intergenic area, and in the Gene column, the mutation is not in the gene area.

For Omicron BA.1, a total of 37 variant-specific nucleotide mutations were identified. A total of 16 of these were nucleotide substitutions, of which 12 led to the mutation of amino acid, and four were synonymous mutations. The rest (n = 21) were nucleotide deletions, and 18 of these were mutations leading to amino acid deletion, while three were associated with amino acid change (Table 2).

A total of 53 Omicron BA.2 variant-specific nucleotide mutations were identified. Of these, 24 were nucleotide substitutions, of which 17 led to amino acid mutation, and seven were synonymous mutations. Twenty-nine nucleotide deletions led to amino acid deletion and one to amino acid change. A total of 17 were intergenic mutations (Table 2).

### Detected SARS-CoV-2 variants in wastewater and their proportions

**Figure 3:**
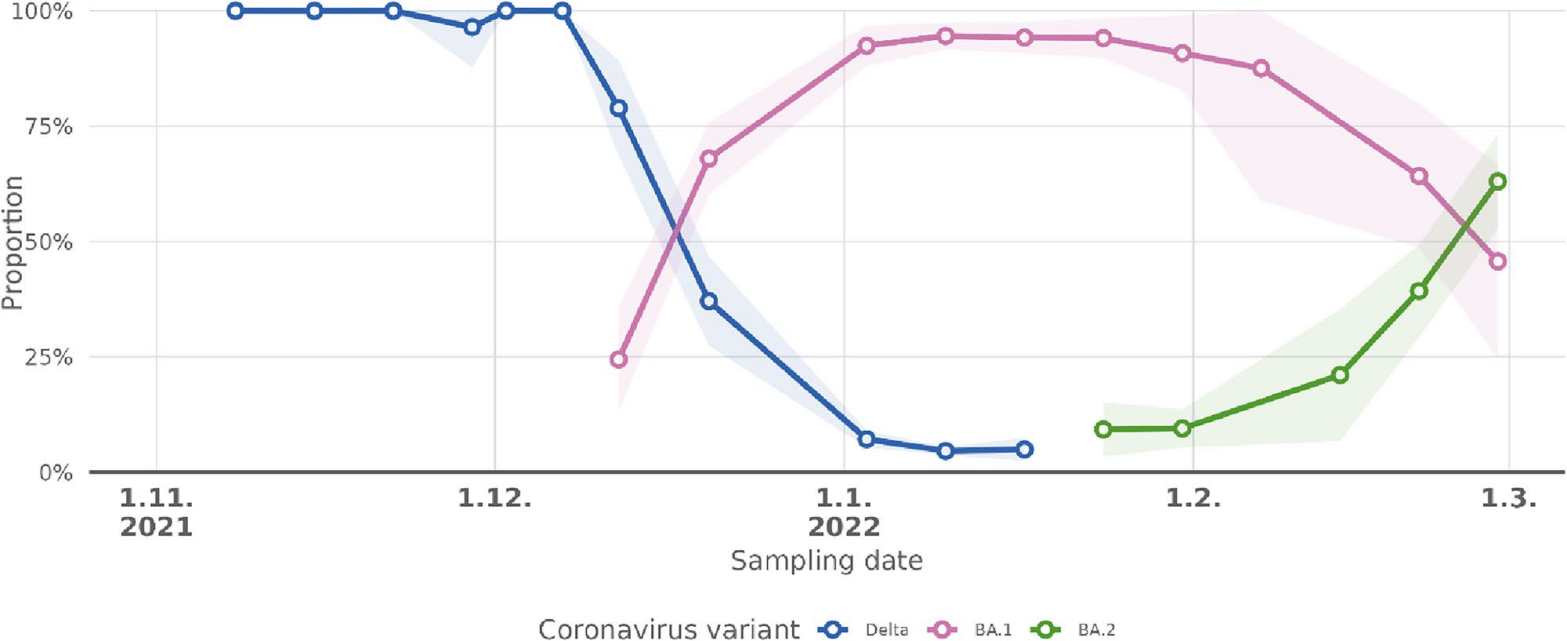
Proportions of the Delta B.1.617.2, Omicron BA.1, and Omicron BA.2 variants in Helsinki wastewater, 8.11.2021 to 28.2.2022. Delta B.1.617.2 was the most common variant until 13.12.2021. Omicron BA.1 became the most common on 20.12.2021. On 28.2.2022, Omicron BA.2 became the most common. The standard deviation of the mutation allelic frequency is in the lighter shaded area.

Based on the detected variant specific mutations, Delta B.1.617.2 was found in wastewater samples from the start of the current study (8.11.2021) until 17.1.2022, according to a hypergeometric test (p < 0.05, Table 3). Delta B.1.617.2 was the most dominant variant in the Helsinki wastewater for the whole year (2022) until 20.12.2022 when Omicron BA.1 became the variant with the highest proportion (p < 0.05) (Fig. 3). Omicron BA.1 was observed for the first time in the influent wastewater of Viikinmäki WWTP jn Helsinki in a composite sample taken on 12–13.12.2021. Omicron BA.2 was observed for the first time on 24.1.2022 (p < 0.05). Both Omicron BA.1 and BA.2 were observed until the end of this study period (28.2.2022). The Omicron BA.2 variant became more common on 28.2.2022 than Omicron BA.1.

**Table 3:** Detected SARS-CoV-2 mutations found in Helsinki, Viikinmäki WWTP wastewater samples between November 2021 and February 2022. The *P*-value of hypergeometric distribution when comparing found mutations to known mutations of the variants in the sample. When the *p*-value of the hypergeometric test was < 0.05, the variant was considered found. Underlining represents synonymous nucleotide mutations.

Sampling start date	Number of all detected mutations	Delta B.1.617.2			Omicron BA.1			Omicron BA.2		
		Number of variant specific mutations 33			Number of variant specific mutations 37			Number of variant specific mutations 53		
		Number of detected variant specific mutations	p-value	Mutations found	Number of detected variant specific mutations	p-value	Mutations found	Number of detected variant specific mutations	p-value	Mutations found
8.11.2021	24	22	< 0.0001	A11332G, A1306S, A1918V, C8986T, D377Y, D63G, D950N, G210T, G215C, I82T, L452R, P1000L, P2287S, P681R, R203M, S26L, T120I, T19R, T3646A, T40I, V2930L, V82A	0	–	–	0	–	–
15.11.2021	23	23	< 0.0001	A11332G, A1306S, A1918V, C8986T, D377Y, D63G, D950N, G210T, G215C, I82T, L452R, P1000L, P2046L, P2287S, P681R, R203M, S26L, T120I, T19R, T3646A, T40I, V2930L, V82A	0	–	–	0	–	–
22.11.2021	24	24	< 0.0001	A11332G, A1306S, A1918V, C8986T, D377Y, D63G, D950N, G210T, G215C, G662S, I82T, L452R, P1000L, P2046L, P2287S, P681R, R203M, S26L, T120I, T19R, T3646A, T40I, V2930L, V82A	0	–	–	0	–	–
29.11.2021	30	22	< 0.0001	A11332G, A1306S, A1918V, C8986T, D377Y, D63G, D950N, G210T, G215C, I82T, L452R, P1000L, P2046L, P2287S, P681R, R203M, T120I, T19R, T3646A, T40I, V2930L, V82A	0	–	–	0	–	–
7.12.2021	25	23	< 0.0001	A11332G, A1306S, A1918V, C8986T, D377Y, D63G, D950N, G210T, G662S, I82T, L452R, P1000L, P2046L, P2287S, P681R, R203M, S26L, T120I, T19R, T3646A, T40I, V2930L, V82A	0	–	–	0	–	–
12.12.2021	45	23	< 0.0001	A11332G, A1306S, A1918V, C8986T, D377Y, D63G, D950N, G210T, G215C, G662S, I82T, L452R, P1000L, P2046L, P2287S, P681R, R203M, T120I, T19R, T3646A, T40I, V2930L, V82A,	13	< 0.0001	A2710T, A67V, C15240T, D3G, G446S, G496S, I3758V, K856R, L981F, N856K, T13195C, T5386G, T547K	1	0.9760	C26858T
20.12.2021	47	24	< 0.0001	A11332G, A1306S, A1918V, C8986T, D377Y, D63G, D950N, G210T, G215C, G662S, I82T, L452R, P1000L, P2046L, P2287S, P681R, R203M, S26L, T120I, T19R, T3646A, T40I, V2930L, V82A	14	< 0.0001	A2710T, A27259C, A67V, C15240T, D3G, G446S, G496S, I3758V, K856R, L981F, N856K, T13195C, T5386G, T547K	0	-	-
3.1.2022	38	21	< 0.0001	A11332G, A1306S, C8986T, D377Y, D63G, D950N, G210T, G215C, I82T, L452R, P1000L, P2046L, P2287S, P681R, R203M, T120I, T19R, T3646A, T40I, V2930L, V82A	13	< 0.0001	A2710T, A67V, C15240T, D3G, G446S, G496S, I3758V, K856R, L981F, N856K, T13195C, T5386G, T547K	0	-	-
10.1.2022	36	15	< 0.0001	A11332G, A1918V, D377Y, D63G, D950N, G215C, I82T, L452R, P2046L, P681R, R203M, S26L, T120I, T40I, V82A	14	< 0.0001	A2710T, A27259C, A67V, C15240T, D3G, G446S, G496S, I3758V, K856R, L981F, N856K, T13195C, T5386G, T547K	0	-	-
17.1.2022	47	15	0.2372	A11332G, A1918V, D377Y, D63G, D950N, G215C, I82T, L452R, P2046L, P681R, R203M, S26L, T120I, T40I, V82A	13	< 0.0001	A2710T, A67V, C15240T, D3G, G446S, G496S, I3758V, K856R, L981F, N856K, T13195C, T5386G, T547K	8	0.3638	C12880T, C15714T, C22792T, D405N, G10447A, G1307S, R408S, S413R
24.1.2022	48	1	0.6245	A1918V	14	< 0.0001	A2710T, A27259C, A67V, C15240T, D3G, G446S, G496S, I3758V, K856R, L981F, N856K, T13195C, T5386G, T547K	17	< 0.0001	A9424G, C10198T, C15714T, C22792T, C26858T, D405N, G10447A, G1307S, L3027F, L3201F, R1315C, R408S, S135R, T223I, T3090I, T842I, V213G
31.1.2022	44	0	–	–	14	< 0.0001	A2710T, A27259C, A67V, C15240T, D3G, G446S, G496S, I3758V, K856R, L981F, N856K, T13195C, T5386G, T547K	13	0.0009	C12880T, C15714T, C22792T, C26858T, D405N, G10447A, G1307S, R408S, S413R, T19I, T223I, T842I, V213G
7.2.2022	32	0	–	–	11	< 0.0001	A2710T, A27259C, A67V, G446S, G496S, I3758V, K856R, L981F, T13195C, T5386G, T547K	4	0.5575	C12880T, C26858T, S413R, T223I
14.2.2022	49	1	0.9699	T40I	6	0.1024	A27259C, G496S, K856R, L981F, T13195C, T547K	14	0.0006	A9424G, C12880T, C26858T, G10447A, L3027F, L3201F, S135R, S413R, T19I, T223I, T3090I, T376A, T842I, V213G
21.2.2022	36	0	–	–	14	< 0.0001	A2710T, A27259C, A67V, C15240T, D3G, G446S, G496S, I3758V, K856R, L981F, N856K, T13195C, T5386G, T547K	19	< 0.0001	A9424G, C10198T, C12880T, C15714T, C22792T, C26858T, D405N, G10447A, G1307S, L3201F, R1315C, R408S, S135R, S413R, T19I, T223I, T3090I, T842I, V213G
28.2.2022	27	0	–	–	11	< 0.0001	A2710T, A27259C, D3G, G446S, G496S, I3758V, K856R, L981F, N856K, T13195C, T547K	16	< 0.0001	C10198T, C12880T, C15714T, C22792T, C26858T, D405N, G10447A, L3201F, R1315C, R408S, S135R, S413R, T19I, T223I, T3090I, T842I

Notably, the Delta variant was detected by hypergeometric tests in the wastewater sample collected on 8.11.2022, even if only 22 of the 33 variant specific mutations were found (p < 0.05). This may be related to 76.9% uniformity and 89.0% coverage, indicating that part of the genome does not have enough reads to assess Delta mutations. This indicates that a hypergeometric test is able to justify the existence of the variant in the sample, even with partial genome coverage (Tables 1 and 3). Interestingly, 13 samples out of 16 contained mutations that were not identified to Delta, Omicron BA.1, or Omicron BA.2 variants (Table 3), indicating that method also identified mutations that are not included in the list of variant specific mutations (Table 1).

## Discussion

The present study introduces a complete protocol to identify variants of the SARS-CoV-2 virus and its proportions in wastewater samples. The protocol was tested with 24 h composite samples from influent derived from Helsinki Viikinmäki Wastewater Treatment Plant, Finland. The composite wastewater samples were concentrated with ultrafiltration columns prior to nucleic acid extraction followed by cDNA synthesis. cDNA was used to generate the ARTIC amplicon library, which was then sequenced with Illumina NGS platform. Bioinformatics of NGS data contained the mapping of reads to the SARS-CoV-2 reference genome with BWA-MEM, following primer trimming, detection of mutations, and calculation of allelic frequencies with iVar following quality control along with uniformity, coverage, and the number of mapped reads. Finally, the existence of the variant was evaluated with hypergeometric distribution following the calculation of variant proportions in the wastewater sample. The protocol presented here was used in Finland to assess virus variants and their proportions in the population, and could also be used to evaluate variants in wastewater elsewhere.

To identify the variant specific mutations, we utilized the CoV-Spectrum platform which contains the percentage proportion of amino acid and nucleotide mutations of the clinical sample sequences submitted to database^18^. The CoV-Spectrum has been used as a source to identify variant specific mutations in order to compare clinical and wastewater data and the identification of variants from wastewater studies^26–28^. This way, we could systematically identify variant specific mutations and use them to recognize SARS-CoV-2 variants from wastewater sequencing data in the time frame of interest and update list of specific mutations to recognize other variants. To extend the pipeline to follow other properties of the virus in the population, mutations which might affect transmission or disease severity could be included in the reference list.

As wastewater is a difficult sample matrix affected by variations in the amount and composition of environmental factors and PCR inhibitors, this results in a situation where part of the target genome may not have sufficient coverage^14^. As the data presented herein shows, the whole genome may not be covered, even when uniformity percentage and genome coverage are at a sufficient level. Our strategy of using two RNA dilutions before cDNA synthesis from the same sample resulted in over 90% genomic coverage and over 85% uniformity on average, indicating a high success rate but also gaps in the sequencing data. In the optimal case, when sequencing reads are evenly distributed to the target genome and have at least 100× coverage, it should be possible to detect a virus proportion of about 3–6%^23^. To evaluate the quality of sequencing, we utilized genome coverage percentage and uniformity as quality parameters. The use of one of these parameters alone could lead to a situation where the sample has sufficient mapped reads, but they are unevenly distributed; or reads are evenly distributed but the coverage is too low to identify mutations and evaluate the allelic frequency.

Similar to our study, many others have used iVar for primer clipping and identification of nucleotide mutations in SARS-CoV-2 wastewater studies^9,23,29–31^. Identified mutations in the samples were systematically categorized into three categories in accordance with the number of mapped reads, sequencing depth, and iVar p-value to evaluate their capability to identify variants and calculate variant proportions in the samples. For the detection of mutations, we used the threshold of 20 mapped reads, as suggested by World Health Organization instructions for clinical samples to detect mutations^25^. Since wastewater samples might contain more diverse mutations than clinical samples^5^, and some errors in sequencing are possible, the use of any lower threshold might lead to the detection of erroneous mutations. However, to calculate allelic frequency and later in evaluating proportions of the variant, we used > 100 reads per mutation as a coverage threshold to ensure sufficient coverage and reliable value for allelic frequency, as previously described by Rios et al*.*^9^. This coverage should be enough to detect a frequency of about 10%. However, higher coverage would reduce variation in the estimation of allelic frequency^23^.

Evaluation of the presence of variants may be difficult if there are gaps in genome coverage and a variation in the number of variant specific mutations, which have been recognized as challenges in variant detection from wastewater^5^. To evaluate the presence of variants in the sample, we used a hypergeometric test, which has been previously used widely in bioinformatics analysis to evaluate, e.g., enriched pathways in gene expression data^32,33^. With this strategy, the evaluation of the presence of variants in the wastewater sample is feasible, even if the sequencing data does not cover all genome areas.

During the period of the study, Delta B.1.617.2, Omicron BA.1, and Omicron BA.2 variants were identified from wastewater influent of Helsinki wastewater. Overall, identified variants and their proportions show positive agreement with clinical samples in the Helsinki area, indicating that this pipeline follows temporal and spatial variation of variants at the population level when visually comparing trends^34^. We also found some mutations that were not on the list of variant-specific mutations. These mutations may be associated with the founder effect of some variant in Finland, or represent traits of new variants in the population^35^. Also, those mutations may be shared with two or more variants, or are mutations which have frequency below 80% and are thus not includedin the list of variant-specific mutations. This indicates that the limitation of our strategy is the identification of novel variants, since variants are now recognized by predefined mutations. Discovering novel variants from wastewater may also be challenging due to several virus variants/types in sample and short sequencing reads^5,12,35^.

To conclude, the pipeline presented herein is suitable for detecting variants of SARS-CoV-2 from wastewater samples to follow spatial and temporal trends in the population. Also, this pipeline could be easily modified to detect variants of some other pathogen, e.g., influenza or the RS virus, when novel amplicon panels are designed with an appropriate tool^36^ and by replacing reference genome and primer clipping files of this protocol with the corresponding primer panel in use.

## Data Availability

The amplicon sequencing data in FASTQ files generated in this publication have been deposited with links to BioProject accession number PRJNA1042787 in the NCBI BioProject database (https://www.ncbi.nlm.nih.gov/bioproject).
